# Evaluation of the efficacy of Capacitive Resistive Monopolar Radiofrequency at 448 kHz in the physiotherapeutic treatment of female dyspareunia

**DOI:** 10.1016/j.conctc.2025.101433

**Published:** 2025-01-10

**Authors:** Anna Abelló Pla, Anna Andreu-Povar, Laura Fabbi, Jordi Esquirol-Caussa, Judith Lleberia-Juanós, Antonio Gil-Moreno, Mireia Coll Omaña

**Affiliations:** a Escoles Universitàries Gimbernat, Universitat Autònoma de Barcelona, Avinguda de La Generalitat 202, 08174, Sant Cugat del Vallès, Barcelona, Spain; b Universitat de Vic, Universitat Central de Catalunya, Facultat de Ciències de La Salut i del Benestar, Carrer de La Sagrada Família 7, Edifici B, 08500, Vic, Barcelona, Spain; c Universitat Autònoma de Barcelona, Departament de Pediatria, d'Obstetrícia i Ginecologia, i Medicina Preventiva i Salut Pública, Avinguda Can Domènech S/N, Edifici M. Campus de La UAB, 08193, Bellaterra, Barcelona, Spain; d Hospital Universitari de La Vall d’Hebron, Servei de Ginecologia i Obstetricia, Passeig de La Vall d’Hebron 119-129, 08035, Barcelona, Spain

**Keywords:** Dyspareunia, Physical therapy modalities, Sexual dysfunction, Physiological, Musculoskeletal manipulations, Pelvic pain

## Abstract

**Background and purpose:**

Dyspareunia is genital pain associated with sexual activity that affects the quality of life of many women. Physiotherapy is a promising, albeit sometimes uncomfortable, option. This study aims to integrate capacitive resistive monopolar radiofrequency (CRMRF) as a complementary therapy. This study aims to evaluate the benefits of combining CRMRF with vaginal manual physiotherapy in young women with dyspareunia.

**Method:**

ology: A randomized, prospective, single-blind clinical trial was conducted. Women aged 18 to 30 with superficial dyspareunia were divided into two groups: an intervention group (GI) receiving CRMRF and manual therapy, and a control group (GC) receiving only manual therapy. Four sessions were conducted, assessing sexual function using the Female Sexual Function Index (FSFI) and the Female Sexual Function Questionnaire (FSM). Data were collected before and after treatment, with a follow-up at three months.

**Results:**

Both groups showed significant improvements in sexual function at the end of treatment. In the GI, improvements were observed in desire, arousal, lubrication, satisfaction, and pain reduction. The GC also showed improvements in these domains. No significant improvements were observed in either group at three months, suggesting that treatment benefits were maintained without additional long-term improvements.

**Conclusion:**

Manual therapy alone and in combination with CRMRF improved sexual function immediately after treatment. No additional improvements were observed at three months, indicating the sustained benefits and emphasizing the need to evaluate maintenance strategies and consider psychological factors.

**Clinical trials registration:**

#NCT5844189.

## 1 Introduction

Dyspareunia is defined as persistent or recurrent genital pain associated with sexual activity, which can occur before, during, or after intercourse [1]. This pain can vary in intensity and location, affecting sexual pleasure and leading to the avoidance of coital activity [2]. It is classified as primary (pain since the first sexual intercourse) or secondary (pain that appears after satisfactory sexual relations), generalized (occurs with all partners) or situational (appears in certain circumstances), and superficial (at the entrance of the vaginal introitus) or deep (during deep penetration) [2]. Dyspareunia and vaginismus are classified by the American Psychiatric Association as genito-pelvic pain/penetration disorders, negatively affecting sexual health and quality of life, and are more common in women [2].

Dyspareunia is prevalent in young women in Europe, with a variation of 13 %–79 % depending on measurement methods. A study in Sweden (2014) reported that 77 % of women aged 18 to 35 experienced pain lasting more than six months [3]. Another study (2009) indicated that 79 % of women aged 13 to 21 experienced pain during sexual intercourse, with 49 % in the last two months [4]. In Belgium (2015), the prevalence was 19.7 % as sexual difficulty and 14.6 % as sexual dysfunction in women aged 16 to 29 [5]. In the United Kingdom (2017), the prevalence in women aged 16 was 34.5 % [6].

Physiotherapy is a promising treatment for dyspareunia, utilizing techniques like endocavitary manual therapy, including massage or trigger point therapy [2]. Perineal massage is especially recommended for pregnant women to improve the elasticity of the perineal muscles [7]. A 2017 study suggested the use of perineal massage to improve dyspareunia caused by pelvic floor muscle tension, concluding that Thiele massage is effective [7]. However, many women describe perineal massage as uncomfortable and painful, although they reported pain reduction and perineal softening after several sessions [8,9].

This study justifies the need to integrate monopolar capacitive resistive radiofrequency (CRMRF) as a complementary therapeutic option, as it offers the advantage of reducing inflammation, improving blood circulation, and promoting muscle relaxation [10,11], which are crucial aspects for addressing the physical causes of pain during sexual intercourse. Additionally, this study contributes to the literature by focusing on the physiological aspects of dyspareunia, thus complementing the traditional approach that also considers the psychological and emotional aspects of the disorder for comprehensive and effective care.

The objective of this study was to analyze the benefits of CRMRF in the treatment of dyspareunia, under the hypothesis that CRMRF combined with manual vaginal physiotherapy improves sexual health in young women with dyspareunia.

## 2 Materials and methods

### 2.1 Study design

This study was designed as a randomized, experimental, analytical, longitudinal, and prospective clinical trial with single-blind treatment application. All study procedures were reviewed and approved by the corresponding ethics committee.

### 2.2 Participants and sample selection

The study included young women aged 18–30 years who reported experiencing pain during sexual intercourse. The sample size was determined using "GRANMO," requiring 21 participants per group, calculated with an alpha risk of 0.05 and a beta risk of 0.20. Inclusion criteria were women aged 18 to 30 with superficial dyspareunia in the last 12 months. Exclusion criteria included the use of antidepressants, pregnancy and breastfeeding, cardiac pathologies, epilepsy, acute inflammatory processes, pacemakers, skin or mucosal infections in the urogenital area, and any condition that would prevent the understanding of informed consent.

### 2.3 Variables

Two main variables were analyzed in this article. The Female Sexual Function Index (FSFI) [12] is widely used to measure quality of life in terms of sexuality. It consists of 19 questions covering six specific areas of female sexual function: desire, subjective arousal, lubrication, orgasm, satisfaction, and pain. Low FSFI scores (total score ≤26) indicate poorer sexual function. The Female Sexual Function Questionnaire (FSM) [13] examines all stages of the female sexual response, including desire, arousal, orgasm, and satisfaction. Additionally, it allows for the description of relevant aspects of partnered sexual activity, such as anticipatory anxiety, initiative, and confidence in communicating preferences.

### 2.4 Intervention and treatment

After selecting the patients, informed consent was presented, and a code was assigned to ensure confidentiality. Patients were randomly assigned to one of two groups: Control Group (CG), which received manual therapy with the radiofrequency device turned off, and Intervention Group (IG), which received manual therapy with active radiofrequency. The groups were identified by colors (blue for IG and yellow for CG) to maintain single blinding. Manual therapy was based on Thiele's perineal massage, with radiofrequency applied using two active electrodes (external capacitive and internal resistive) and a passive electrode on the back. Four sessions were conducted, one per week, with data collected before and after the sessions, and a retest at three months.

The treatment was carried out using an Intradermik device (Rös's Estética), with a frequency of 448 kHz, 200 W resistive output, and 450 VA capacitive output, ultrasound gel (Clear Ultrasound Gel, OXD Professional Care, REF US-C1), and two types of electrodes: a capacitive electrode (30 mm diameter, flat, for personal use) and a resistive electrode (stainless steel, cylindrical, 8 cm in length, 1.5 cm in diameter, with a screw-on plastic cap to avoid heat concentration). The application temperature was measured using a Thermal Sensory Scale (TSS) (0–9), where 0 is the absence of temperature and 9 is the maximum tolerable before burning.

The treatment included four steps.

1. Application of the capacitive electrode for 3 min (1 % power, TSS scale 0–2) externally over the urogenital triangle and translabially.
2. Direct application of the intracavitary resistive electrode for 5 min (25 % power, TSS scale 5–6).
3. Application of the capacitive electrode for 5 min (8–12 % power, TSS scale 5–6) while performing Thiele's massage.
4. Application of the capacitive electrode for 3 min (1 % power, TSS scale 0–2), as in step 1.

### 2.5 Data collection protocol

During the first visit, patients completed a structured interview and an initial examination. After filling out the initial questionnaire, they signed the informed consent for intracavitary physiotherapy. The examination involved manual palpation and pressure evaluation using an Epi-NO device to detect discomfort thresholds. Participants were assigned unique codes for data collection and given a tablet to complete the FSFI and FSM questionnaires. During the final visit and retest, a structured interview was conducted, and the tablet was provided to complete the FSFI and FSM questionnaires. The intracavitary examination was performed according to the initial protocol.

### 2.6 Statistical analysis

SPSS Statistics (v20) was used for statistical analysis, including normality tests, descriptive analysis, and mean comparisons within and between groups. Statistical significance was set at p < 0.05.

### 2.7 Bioethical requirements and confidentiality

The study was approved by the Animal and Human Experimentation Ethics Committee of the Universitat Autònoma de Barcelona (CEEAH-UAB), project number: 5671. The study adhered to Good Clinical Practice guidelines, the Declaration of Helsinki, and applicable legal regulations. All researchers signed a certificate of ethical commitment. Strict confidentiality standards were ensured according to Regulation 2016/679 and Organic Law March 3/2018. Participant data were coded and only accessible to authorized personnel.

## 3 Results

### 3.1 FSFI total score analysis at treatment conclusion

Significant improvements were noted in various aspects of the FSFI total score at the conclusion of treatment (see Table 1, Table 2) in both the Intervention Group (IG) and Control Group (CG). In the IG, significant improvements were found in the domains of desire (p = 0.003), arousal (p = 0.001), lubrication (p = 0.002), satisfaction (p = 0.002), and pain (p = 0.002). The CG also showed significant improvements in desire (p = 0.028), arousal (p = 0.004), lubrication (p = 0.001), satisfaction (p = 0.006), and pain (p < 0.001).

**Table 1:** FSFI results post-treatment.

Contrast statistics^a^								
FSFI		Desire	Excitement	Lubrication	Orgasm	Satisfaction	Pain	Total Score
**IG**	**Z**	−2.991^b^	−3256^b^	−3091^b^	−2819^c^	−3088^b^	−3062^b^	−3827^b^
	**Asymptotic bilateral Sig.**	0,003	0,001	0,002	0,005	0,002	0,002	0,000
**CG**	**Z**	−2194^b^	−2915^b^	−3220^b^	−2462^c^	−2724^b^	−3935^b^	−3696^b^
	**Asymptotic bilateral Sig.**	0,028	0,004	0,001	0,014	0,006	0,0	0

a) Wilcoxon signed-rank test.

b) Based on positive ranks.

c) Based on negative ranks.

**Table 2:** FSFI results after 3 months.

Contrast statistics^a^								
FSFI		Desire	Excitement	Lubrication	Orgasm	Satisfaction	Pain	Total Score
**IG**	**Z**	−1,511^b^	−0,775^b^	−0,418^c^	−0,758^c^	−0,255^c^	−0,686^c^	−0,224^b^
	**Asymptotic bilateral Sig.**	0,131	0,438	0,676	0,448	0,798	0,487	0,823
**CG**	**Z**	−0,081^b^	−0,087^b^	−0,756^c^	−0,235^b^	−0,821^c^	−0,777^c^	−0,785^c^
	**Asymptotic bilateral Sig.**	0,935	0,931	0,450	0,814	0,411	0,437	0,433

a) Wilcoxon signed-rank test.

b) Based on positive ranks.

c) Based on negative ranks.

### 3.2 Post-treatment evaluation at 3 months

Three months post-treatment, neither group exhibited significant changes. In the IG, none of the improvements in the FSFI domains were statistically significant. Scores for desire, arousal, lubrication, orgasm, satisfaction, and pain showed no significant changes, with p-values ranging from 0.131 to 0.798. The CG exhibited a similar trend, with none of the improvements being statistically significant, with p-values between 0.411 and 0.935.

### 3.3 FSM total score analysis at treatment conclusion

The analysis of the Female Sexual Function Questionnaire (FSM) total score at the end of the treatment (see Table 3, Table 4) also indicated significant improvements in several domains within the IG, including arousal (p = 0.001), lubrication (p = 0.007), vaginal penetration problems (p = 0.040), anticipatory anxiety (p = 0.005), initiative (p = 0.004), and degree of communication (p = 0.026). In the CG, significant improvements were observed in vaginal penetration problems (p = 0.056), anticipatory anxiety (p = 0.052), and satisfaction with sexual activity (p = 0.014).

**Table 3:** FSM results post-treatment.

Contrast statistics^a^											
FSM		Desire	Excitement	Lubrication	Orgasm	Problems with Vaginal Penetration	Anticipatory Anxiety	Initiative	Degree of Communication	Satisfaction with Sexual Activity	General Sexual Satisfaction
**IG**	**Z**	−3,0^b^	−3263^b^	−2684^b^	−1112^b^	−2059^b^	−2809^b^	−2887^b^	−2228^b^	−2364^b^	−0,710^b^
	**Asymptotic bilateral Sig.**	0,003	0,001	0,007	0,266	0,040	0,005	0,004	0,026	0,018	0,478
**CG**	**Z**	−0,970^b^	−1390^b^	−1155^b^	−1081^b^	−0,114^b^	−1910^b^	−1941^b^	−1063^b^	−2463^b^	−1955^b^
	**Asymptotic bilateral Sig.**	0,322	0,165	0,248	0,279	0,149	0,056	0,052	0,288	0,014	0,051

c) Based on negative ranks.

a) Wilcoxon signed-rank test.

b) Based on positive ranks.

**Table 4:** FSM results after 3 months.

Contrast statistics^a^											
FSM		Desire	Excitement	Lubrication	Orgasm	Problems with Vaginal Penetration	Anticipatory Anxiety	Initiative	Degree of Communication	Satisfaction with Sexual Activity	General Sexual Satisfaction
**IG**	**Z**	−0,80^b^	−0,802^b^	−0,577^b^	−1406^b^	−1753^b^	−1613^b^	0,00^c^	−0,816^b^	−1217^b^	−0,811^b^
	**Asymptotic bilateral Sig.**	0,936	0,422	0,564	0,160	0,080	0,107	1	0,414	0,223	0,417
**Grupo amarrillo CG**	**Z**	−0,457^b^	−0,546^b^	−0,264^b^	−1134^b^	−0,812^b^	−1414^b^	−0,302^b^	−1^c^	−0,796^b^	−2236^b^
	**Asymptotic bilateral Sig.**	0,648	0,585	0,792	0,257	0,417	0,157	0,763	0,317	0,426	0,025

a) Wilcoxon signed-rank test.

b) Based on positive ranks.

c) Based on negative ranks.

### 3.4 Post-treatment evaluation at 3 months

At the three-month follow-up, the IG showed no significant improvements in any FSM domain, with p-values ranging from 0.080 to 0.936. In the CG, only general sexual satisfaction showed a significant improvement (p = 0.025).

### 3.5 Pre-treatment vs. post-treatment comparison at 3 months

A comparison of the results before the treatment and three months after its conclusion using the FSFI test (see Table 5) showed significant improvements in the IG across the domains of desire (p = 0.001), arousal (p < 0.001), lubrication (p = 0.008), orgasm (p = 0.017), satisfaction (p = 0.004), pain (p = 0.004), and total score (p < 0.001). In the CG, significant improvements were observed in the domains of desire (p = 0.024), arousal (p = 0.018), lubrication (p = 0.024), orgasm (p = 0.032), pain (p < 0.001), and total score (p = 0.003), but not in satisfaction (p = 0.198).

**Table 5:** Comparison of initial FSFI vs. after 3 months.

Contrast statistics^a^								
FSFI		Desire	Excitement	Lubrication	Orgasm	Satisfaction	Pain	Total Score
**IG**	**Z**	−3203^b^	−3295^b^	−2661^b^	−2398^b^	−2905^b^	−2900^b^	−3763^b^
	**Asymptotic bilateral Sig.**	0,001	<0,001	0,008	0,017	0,004	0,004	<0,001
**CG**	**Z**	−2260^b^	−2356^b^	−2254^b^	−2139^b^	−1288^b^	−3370^b^	−2940^b^
	**Asymptotic bilateral Sig.**	0,024	0,018	0,024	0,032	0,198	<0,001	0,003

a )Wilcoxon signed-rank test.

b )Based on negative ranks.

In the FSM questionnaire (see Table 6), significant differences were observed in the IG in the domains of desire (p = 0.003), arousal (p = 0.004), lubrication (p = 0.021), orgasm (p = 0.026), vaginal penetration problems (p = 0.04), anticipatory anxiety (p = 0.001), initiative (p = 0.021), degree of communication (p = 0.013), and satisfaction with sexual activity (p = 0.005), but not in general sexual satisfaction (p = 0.118). In the CG, significant differences were found in the domains of orgasm (p = 0.017), vaginal penetration problems (p = 0.017), anticipatory anxiety (p = 0.005), and satisfaction with sexual activity (p = 0.004), but not in desire (p = 0.471), arousal (p = 0.068), lubrication (p = 0.160), initiative (p = 0.096), or degree of communication (p = 0.524).

**Table 6:** Comparison of initial FSM vs. after 3 months.

Contrast statistics^a^											
FSM		Desire	Excitement	Lubrication	Orgasm	Problems with Vaginal Penetration	Anticipatory Anxiety	Initiative	Degree of Communication	Satisfaction with Sexual Activity	General Sexual Satisfaction
**IG**	**Z**	−2533^b^	−2863^b^	−2305^b^	−2230^b^	−2845^b^	−3211^b^	−2308^b^	−2484^b^	−2812^b^	−1565^b^
	**Asymptotic bilateral Sig.**	0,003	0,004	0,021	0,026	0,04	0,001	0,021	0,013	0,005	0,118
**CG**	**Z**	−0,720^b^	−1827^b^	−1406^b^	−2460^b^	−2390^b^	−2818^b^	−1667^b^	−0,638^b^	−2914^b^	−2443^b^
	**Asymptotic bilateral Sig.**	0,471	0,068	0,160	0,014	0,017	0,005	0,096	0,524	0,004	0,015

a Wilcoxon signed-rank test.

b Based on negative ranks.

## 4 Discussion

The study results show that both the Intervention Group (IG) and the Control Group (CG) achieved significant improvements in sexual function immediately after treatment, as evidenced by the scores from the Female Sexual Function Index (FSFI) and the Female Sexual Function Questionnaire (FSM). No significant improvements were observed at the 3-month follow-up, suggesting that the improvements achieved were maintained over time.

The lack of significance in the follow-up at 3 months suggests that the beneficial effects of manual therapy and capacitive resistive monopolar radiofrequency (CRMRF) present immediately after treatment can be maintained in the long term, albeit without additional improvements. This finding is consistent with previous studies that have observed a decline in therapeutic effects over time in dyspareunia treatments. For instance, a study reported initial improvements in sexual function that were not maintained at a 6-month post-intervention follow-up, highlighting the need for periodic interventions to sustain therapeutic benefits [14].

These findings highlight the importance of assessing and refining post-treatment maintenance strategies to extend benefits. One approach that could be beneficial is the integration of reinforcement therapies, such as monthly maintenance sessions, which could help maintain the benefits obtained immediately after the initial treatment. Research has suggested that implementing these sessions can prolong the positive effects of dyspareunia treatment [15].

It is noteworthy that the CG, which received only manual therapy, also showed significant improvements in several domains, suggesting that manual therapy alone has a positive impact on dyspareunia. Manual therapy has been extensively studied and has shown that it can be effective in improving sexual function and reducing pain in women with dyspareunia [[15], [16], [17], [18], [19], [20]].

Comparing the results obtained before starting the treatment with the evaluation conducted 3 months post-treatment, we observe different outcomes in the FSM questionnaire. The group treated with CRMRF showed significant improvements in all domains except general sexual satisfaction, indicating the possibility of interaction with external psychological factors beyond the treatment. In the CG, significant improvements were observed only in the domains of orgasm, vaginal penetration problems, anticipatory anxiety, and sexual satisfaction. The isolated application of manual therapy improved the physical aspects of dyspareunia and some associated psychological aspects; as pain decreases, anxiety reduces, and satisfaction increases [18].

Although some of the results presented might suggest that the use of CRMRF does not add additional benefits to the treatment regarding sexual function, other aspects such as pain and discomfort during the application of treatment, which showed differences, have been evaluated and have been presented in another article [21]. The analysis of the size of the effect using Cohen's d provides an additional perspective on the effectiveness of the intervention. After treatment, the d value was positive (p = 0.26), favoring the intervention group, although the size of the effect was small. At three months, the value remained positive and moderate (d = 0.45), suggesting that the benefits of the intervention were consolidated over time. This pattern of results indicates that, although the control group showed improvements, the intervention group experienced an effect more noticeable as time went on. This suggests that the intervention was effective not only in the short term but also had a lasting impact on the participants' sexual function.

Psychological factors likely influence the sustainability of post-treatment improvements. Emotional state, pain perception, and sexual satisfaction are highly susceptible to psychological influences. Research has demonstrated that anxiety, depression, and stress negatively affect sexual function and pain perception [22]. In the context of dyspareunia, psychological factors can play a crucial role in pain perception and sexual satisfaction. One study found that high levels of anxiety and stress were associated with greater persistence of pain in women with dyspareunia [23]. Female sexual function is a complex, multidimensional construct encompassing more than physiological indicators [24,25]. Its understanding requires an approach that incorporates the physical and psychological, emotional and social aspects [26], a possible limitation in this study since only the physical and physiological aspect of dyspareunia has been assessed and treated.

The findings highlight the importance of further exploring treatment combinations and therapy duration to maximize long-term outcomes in managing dyspareunia. The effects obtained after the intervention, although not always statistically significant compared to the control group, reveal that the CRMRF intervention has a positive and sustained impact on muscle function recovery and pain reduction, suggesting its usefulness as a complement to manual therapy, especially to help prepare the tissues for the subsequent manual therapy.

## 5 Conclusions

This study evaluated the efficacy of CRMRF in combination with manual therapy for the treatment of female dyspareunia, compared to manual therapy alone. The results obtained allow for several significant conclusions.

Firstly, it was observed that both the Intervention Group, which received manual therapy with CRMRF, and the Control Group, which received only manual therapy, showed significant improvements in sexual function immediately after treatment.

At three months post-treatment, no significant improvements were observed. This suggests that the beneficial effects of manual therapy and CRMRF, present immediately after treatment, were maintained in the long term without the provision of a maintenance strategy.

Nevertheless, we emphasize the need to evaluate and possibly adjust post-treatment maintenance strategies to help sustain the positive effects of the initial treatment. Furthermore, it is important to consider the influence of psychological factors on the sustainability of post-treatment improvements, as emotional state and pain perception can significantly affect sexual function.

## CRediT authorship contribution statement

**Anna Abelló Pla:** Writing – review & editing, Writing – original draft, Validation, Supervision, Methodology, Investigation, Conceptualization. **Anna Andreu-Povar:** Writing – original draft, Methodology, Investigation, Formal analysis, Data curation, Conceptualization. **Laura Fabbi:** Writing – review & editing, Investigation. **Jordi Esquirol-Caussa:** Writing – review & editing, Validation, Supervision, Project administration, Methodology, Formal analysis, Conceptualization. **Judith Lleberia-Juanós:** Writing – review & editing, Validation, Supervision. **Antonio Gil-Moreno:** Writing – review & editing, Validation, Supervision. **Mireia Coll Omaña:** Writing – review & editing, Investigation.

## Patient consent statement

This study involved human participants, and written informed consent was obtained from all individuals included in the study. Participants were provided with detailed information regarding the study's objectives, procedures, potential risks, and benefits. They were assured that their participation was entirely voluntary and that they had the right to withdraw from the study at any time. Participants were informed that their data would be used in scientific publications and presentations. They understood that their personal information would be kept confidential, and any identifying details would be anonymized in any publication arising from this study.

## Data availability statement

The data supporting the findings of this study are available from the corresponding author upon reasonable request.

## Disclosure of interests

The authors do not have any interests to disclose.

## Ethics of approval statement

This study was conducted in accordance with the ethical standards outlined in the Declaration of Helsinki and was approved by the Ethics Committee for Animal and Human Experimentation (CEEAH-UAB) at Universitat Autònoma de Barcelona. The protocol number for this study is 5671.

## Funding statement

The authors report there was no funding to declare.

## Declaration of competing interest

The authors declare that they have no known competing financial interests or personal relationships that could have appeared to influence the work reported in this paper.
